# Sutimlimab improves quality of life in patients with cold agglutinin disease: results of patient-reported outcomes from the CARDINAL study

**DOI:** 10.1007/s00277-022-04948-y

**Published:** 2022-08-23

**Authors:** Alexander Röth, Wilma Barcellini, Tor Henrik Anderson Tvedt, Yoshitaka Miyakawa, David J. Kuter, Jun Su, Xiaoyu Jiang, William Hobbs, Jaime Morales Arias, Frank Shafer, Ilene C. Weitz

**Affiliations:** 1 Department of Hematology and Stem Cell Transplantation, West German Cancer Center, University Hospital Essen, University of Duisburg-Essen, Essen, Germany; 2 Fondazione IRCCS Ca’ Granda Ospedale Maggiore Policlinico, Milan, Italy; 3 Section for Hematology, Department of Medicine, Haukeland University Hospital, Bergen, Norway; 4 Department of General Internal Medicine, Saitama Medical University Hospital, Saitama, Japan; 5 Division of Hematology, Massachusetts General Hospital, Harvard Medical School, Boston, MA USA; 6 Sanofi, Cambridge, MA USA; 7 Jane Anne Nohl Division of Hematology, Department of Medicine, University of Southern California – Keck School of Medicine, Los Angeles, CA USA

**Keywords:** Cold agglutinin disease, Sutimlimab, Functional Assessment of Chronic Illness Therapy-Fatigue, Patient-reported outcome, Quality of life

## Abstract

Patients with cold agglutinin disease (CAD) experience fatigue and poor quality of life. However, previous CAD-related studies have not explored patient-reported outcomes such as the Functional Assessment of Chronic Illness Therapy (FACIT)-Fatigue. Sutimlimab, a C1s complement inhibitor, has been shown to halt haemolysis in CAD. Here, we present 26-weeks’ patient-reported data from CARDINAL Part A (ClinicalTrials.gov, NCT03347396), which assessed efficacy and safety of sutimlimab in patients with CAD and recent history of transfusion. Aside from measuring changes in haemolytic markers, FACIT-Fatigue was measured at the treatment assessment timepoint (TAT; average of weeks 23, 25, and 26). Exploratory endpoints included the change in EuroQol 5-dimension 5-level questionnaire (EQ-5D-5L) and the 12-Item Short Form Health Survey (SF-12) at TAT, and Patient Global Impression of Change (PGIC), and Patient Global Impression of (fatigue) Severity (PGIS) at week 26. Mean (range) FACIT-Fatigue scores increased from 32.5 (14.0–47.0) at baseline (a score indicative of severe fatigue) to 44.3 (28.0–51.0) at TAT. Considerable improvements were reported for EQ-5D-5L at TAT, SF-12 scores at TAT, and PGIC and PGIS scores at week 26. Sutimlimab treatment resulted in sustained improvements in symptoms of fatigue and overall quality of life in patients with CAD. NCT03347396. Registered 20 November, 2017.

**Supplementary Information:**

The online version contains supplementary material available at 10.1007/s00277-022-04948-y.

## Introduction

Cold agglutinin disease (CAD) is a rare autoimmune haemolytic anaemia in which activation of the classical complement pathway (CP) causes chronic haemolysis [1]. Cold agglutinins (CA) are typically immunoglobulin M antibodies that bind to I antigen on the surface of red blood cells, causing agglutination at acral body temperatures [2]. Binding of CAs to C1 complexes causes classical CP activation, resulting in macrophage-based extravascular haemolysis in the liver [1]. Minimal intravascular haemolysis (using C5b-C9) occurs, given intact CD55- and CD59-mediated regulation [1].

CAD impacts quality of life (QOL), with fatigue comparable to chronic illnesses such as cancer and other haemolytic anaemias (e.g. paroxysmal nocturnal haemoglobinuria) [3–5]. Fatigue in CAD may be driven by chronic haemolytic anaemia [6, 7] as well as complement-mediated inflammation [8]. The disease most commonly presents in the seventh decade of life, with a median age at symptom onset between 66 and 72 years, exacerbating the impact of aging and accumulated co-morbidities [7]. Correlations between treatment-induced improvements in haemolysis or complement inhibition and changes to patient-reported QOL remain poorly understood, while prospective studies in CAD rarely include patient-reported outcomes (PRO) [9–12].

Sutimlimab (formerly BIVV009) is a humanised monoclonal IgG4 anti-C1s monoclonal antibody that inhibits the classical CP, while preserving the alternative and lectin-mediated CPs to allow continued pathogen surveillance. In the pivotal, phase 3 single-arm, multicentre CARDINAL study, patients with a recent transfusion history who received 26 weeks’ sutimlimab treatment had rapidly normalised bilirubin levels, increased haemoglobin levels, and remained transfusion-free throughout most of the study [4]. In the phase 3, randomised, double-blind, placebo-controlled CADENZA study, patients with no recent transfusion history who received 26 weeks’ sutimlimab had rapid and sustained significant improvements in anaemia and haemolysis, compared with patients who received placebo [13]. In both the CARDINAL and CADENZA studies, onset of efficacy coincided with a near-complete inhibition of classical complement pathway activity [4, 13]. Patients treated with sutimlimab in the CARDINAL and CADENZA studies also achieved clinically meaningful reductions in fatigue [4, 13, 14]. The objective of the present study was to analyse further the PROs from the pivotal CARDINAL study in patients with CAD and a recent history of transfusion.

## Methods

### Study design and patient population

Full details of the CARDINAL study design and clinical results have already been published [4]. Briefly, CARDINAL was a prospective, open-label, single-arm, multicentre study with two parts (A and B) evaluating sutimlimab in patients with primary CAD and a recent history of transfusion. The primary objective in part A was to assess if treatment with sutimlimab could increase haemoglobin levels by ≥2 g/dL from baseline or normalise haemoglobin levels to ≥12 g/dL while preventing the need for blood transfusion during treatment. Secondary efficacy objectives included evaluating the effect of sutimlimab on QOL (assessed using PROs).

Patients underwent a 6-week screening period and followed by a 26-week treatment period during which sutimlimab, dosed according to body weight, was infused intravenously on days 0 and 7, and biweekly thereafter through week 25. Patients successfully completing the 26-week treatment period could continue to receive sutimlimab in the open-label 2-year extension study (part B), assessing long-term efficacy, safety, and durability of response.

The study was conducted in accordance with consensus ethics principles derived from international ethics guidelines. Patients gave written informed consent for their participation in the study. Eligible patients were ≥18 years of age with a body weight of ≥39 kg, had confirmed primary CAD, and a recent history of transfusion (within 6 months of enrolment). Eligibility criteria also included baseline haemoglobin ≤10 g/dL, active haemolysis (total bilirubin above normal), and the presence of ≥1 CAD-related symptom.

### Endpoints

The secondary outcome measures included mean change from baseline to the treatment assessment timepoint (TAT) in Functional Assessment of Chronic Illness Therapy (FACIT)-Fatigue score assessed at all scheduled visits. FACIT-Fatigue is a 13-item QOL assessment tool, wherein patients evaluate the extent to which they feel fatigued by rating their agreement with 13 items on a 5-point scale (0–4, from ‘not at all’ to ‘very much so’) [15]. The scores for each question are added together to give an overall value (maximum of 52). Items are reverse scored where appropriate, so that higher scores represent better functioning and less severe fatigue [15].

Exploratory QOL endpoints included mean change from baseline to the TAT in the EuroQol 5-dimension 5-level questionnaire (EQ-5D-5L; assessed at days 49, 91, 133 and 182) and the 12-Item Short Form Health Survey (SF-12; assessed at days 35, 77, 133 and 182). Patient Global Impression of Change (PGIC) and Patient Global Impression of (fatigue) Severity (PGIS) were also assessed at days 35, 77, 133 and 182. Patients with CAD-related symptoms were also asked to report on the incidence of these at each visit.

The EQ-5D-5L questionnaire comprises five domains (mobility, self-care, usual activities, pain/discomfort and anxiety/depression) [16]. Patients must select the extent to which their disease impacts each domain on a five-tier scale (from ‘no problem’ to ‘extreme problem’; 0–100). The EQ-5D-5L also contains a visual analogue scale (VAS; 0–100) of perceived overall health [16]. SF-12 is a 12-item questionnaire measuring eight health domains, and is categorised into physical (general health, physical functioning, role physical and body pain) and mental (vitality, social functioning, role emotional and mental health) component scores (PCS and MCS scores are on a T-metric, with mean=50 and SD=10, referenced to the US general population) [17, 18]. Total PCS and MCS are calculated as weighted means of the component domains, where a higher score represents improved QOL [17].

PGIC is a single-question, 7-point scale where patients provide their perception of overall status change from ‘very much improved’ to ‘very much worse’. PGIS is a 5-point scale, where patients’ perception of fatigue severity is measured from ‘very severe’ to ‘no change’ [19].

### Statistical analysis

Sample size was determined assuming a true responder rate of 66%, where a minimum of 30% was required for success. With a sample size of 20 patients, this equated to a 90% probability that the lower limit of the 95% confidence interval for the primary endpoint would be ≥30%. Categorical variables were presented as n (%) for each category, and continuous variables were summarised by descriptive statistics. Baseline was defined as the last value observed during screening. Statistical analysis was performed using SAS version 9.3 (SAS Institute Inc., Cary, NC, USA) or higher. Secondary endpoints were analysed using the mixed model for repeated measures (MMRM) at the TAT, and exploratory PROs were analysed on the full analysis set using descriptive statistics.

## Results

### Baseline demographics

The full analysis set comprised 24 patients from 16 sites in eight countries who received ≥1 dose of sutimlimab. At baseline, enrolled patients were generally female (62.5%) and had a mean (range) age of 71 (55–85) years. The majority of patients had evidence of symptomatic anaemia and other CAD-related disease characteristics (Table 1). Mean (range) FACIT-Fatigue score at baseline was 33 (14–47), and SF-12 PCS and MCS were 38.7 (19.9–52.2) and 49.8 (33.8–63.2), respectively. Mean (range) baseline EQ-5D-5L index and VAS scores were 0.70 (−0.03 to 1.00) and 62.0 (25.0–80.0), respectively. Of the 24 patients enrolled in the study, 91.7% had at least one ongoing/resolved medical history, the most common being surgical and medical procedures (50.0%), cardiac disorders, infections and infestations, and gastrointestinal disorders (all 45.8%). The complete patient baseline demographic and disease characteristics are covered elsewhere [4].

**Table 1:** Baseline patient characteristics (full analysis set)

Mean (range), unless otherwise specified	Total (*N *= 24)
Incidence of symptomatic anaemia, n (%)	
Fatigue	18 (75.0)
Weakness	15 (62.5)
Shortness of breath	13 (54.2)
Palpitations	7 (29.2)
Light headedness	0 (0.0)
Chest pain	2 (8.3)
Other CAD-related disease characteristics, n (%)	
Acrocyanosis	3 (12.5)
Raynaud’s syndrome	1 (4.2)
Haemoglobinuria	5 (20.8)
Disabling circulatory symptoms	2 (8.3)
Major adverse vascular event (including thrombosis)	0 (0.0)
FACIT-Fatigue score	32.5 (14.0–47.0)
SF-12 score, n	22
PCS	38.69 (19.9–52.2)
MCS	49.83 (33.8–63.2)
Total C4, g/L^a^	0.04 (0.0–0.3)
Classical CP, %^b^	20.0 (0.0–55.7)

*CAD*, cold agglutinin disease; *CP*, complement pathway; *EQ-5D-5L*, EuroQol 5-dimension 5-level questionnaire; *FACIT*, Functional Assessment of Chronic Illness Therapy; *MCS*, mental component score; *PCS*, physical component score; *PGIS*, Patient Global Impression of Severity; *SF-12*, 12-Item Short Form Health Survey; *VAS*, visual analogue scale

^a^Normal range for total C4 was 0.18–0.45 g/L. ^b^Classical CP activation was measured using an enzyme-linked immunosorbent assay that measures the functional capacity of the classical CP (Wieslab-classical CP assay) and the normal range is 69–129% (is a statistical calculation and does not guarantee a true cut-off) of the positive control, based on 120 sera from healthy blood donors

### Patient-reported outcome measures

#### FACIT-Fatigue score

**Fig. 1:**
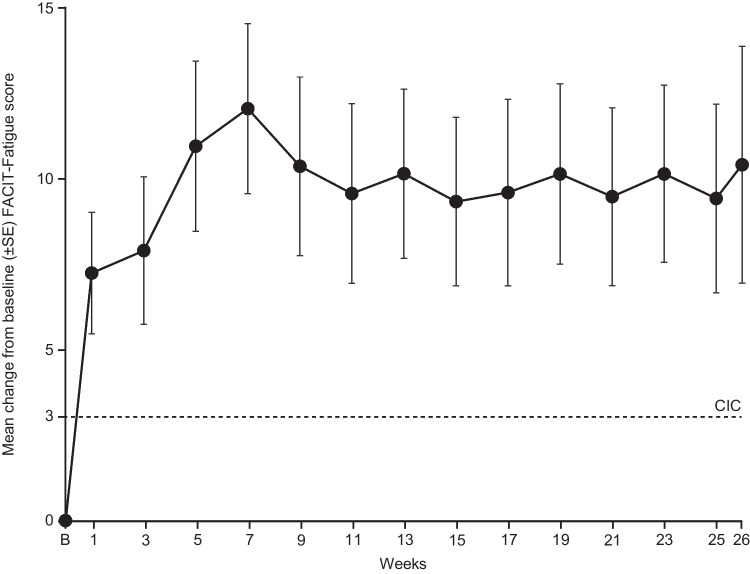
FACIT-Fatigue score through week 26. B, baseline; CIC, clinically important change; FACIT, Functional Assessment of Chronic Illness Therapy; SE, standard error. FACIT-Fatigue Scale scores range from 0 to 52, with a higher score indicating less fatigue. In this study, a score change of ≥ 3 points was considered clinically meaningful

Overall, the mean FACIT-Fatigue score increased rapidly during sutimlimab treatment, indicating improvement in symptoms of fatigue (Fig. 1) and ≥75% of patients achieved an increase in FACIT-Fatigue score of ≥3 points at the TAT (interquartile range, 5.00–15.50 points). Mean (range) FACIT-Fatigue score improved early (week 1) and peaked at week 7 by 12.05 (−3.0 to 34.0; *n*=19) points; improvements were sustained through week 26. Of three patients classified as haematologic non-responders, two showed minimal increases in FACIT-Fatigue score (<1 point) and one decreased compared to baseline (Supplementary Table 1). One patient who missed an infusion (withdrawal of sutimlimab for 28 days) resulted in a FACIT-Fatigue score decline of 44%, from 43 on day 150 to 24 on day 178. This decrease in their FACIT-Fatigue score correlated with an observed rise in CP activity from 4.6% at day 150 to 73% at day 178, with a concomitant drop in C4 levels.

Improvements from baseline to the end of treatment (week 26) were observed in all 13 FACIT-Fatigue component domains (Supplementary Fig. 1; Supplementary Table 2). The substantial improvements in these domains were observed rapidly (week 1) and sustained to week 26.

#### SF-12 PCS and MCS

**Fig. 2:**
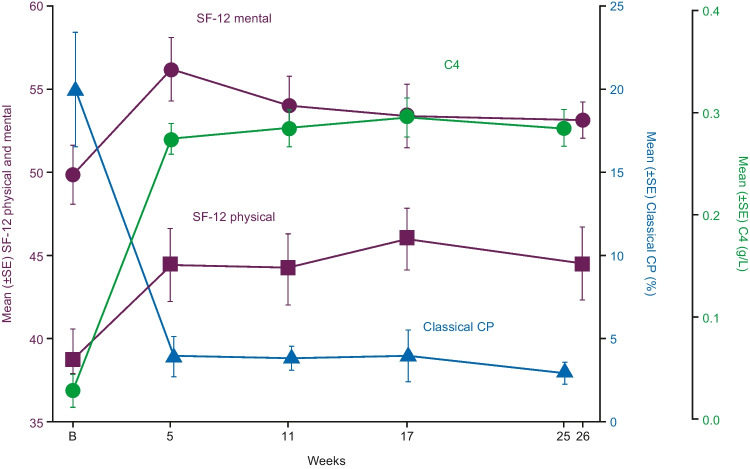
Mean SF-12 physical and mental domain scores versus mean classical CP and total C4 from baseline to week 26 of sutimlimab treatment. B, baseline; CP, complement pathway; SE, standard error; SF-12, 12-Item Short Form Health Survey

By week 5, mean (range) SF-12 PCS and MCS scores increased from 38.7 (20.0–52.2) and 49.8 (33.8–63.2) (*n*=22) to 44.4 (23.4–56.4) and 56.2 (36.6–67.9), respectively (*n*=19). These clinically meaningful improvements were sustained to week 26, where mean (range) change from baseline were 5.37 (−7.0 to 16.0) and 4.47 (−8.1 to 33.2) points to 44.5 (30.2–58.6) and 53.1 (39.5–67.0), respectively (*n*=16). SF-12 score increases were associated with a reduction in classical CP activity and increased total C4 (Fig. 2), which coincided with increases in haemoglobin and normalisation of bilirubin (see CP and C4 results presented in Rӧth et al. [4]). Changes in each SF-12 component and subscale are given in Supplementary Table 3.

#### EQ-5D-5L index and VAS scores

**Fig. 3:**
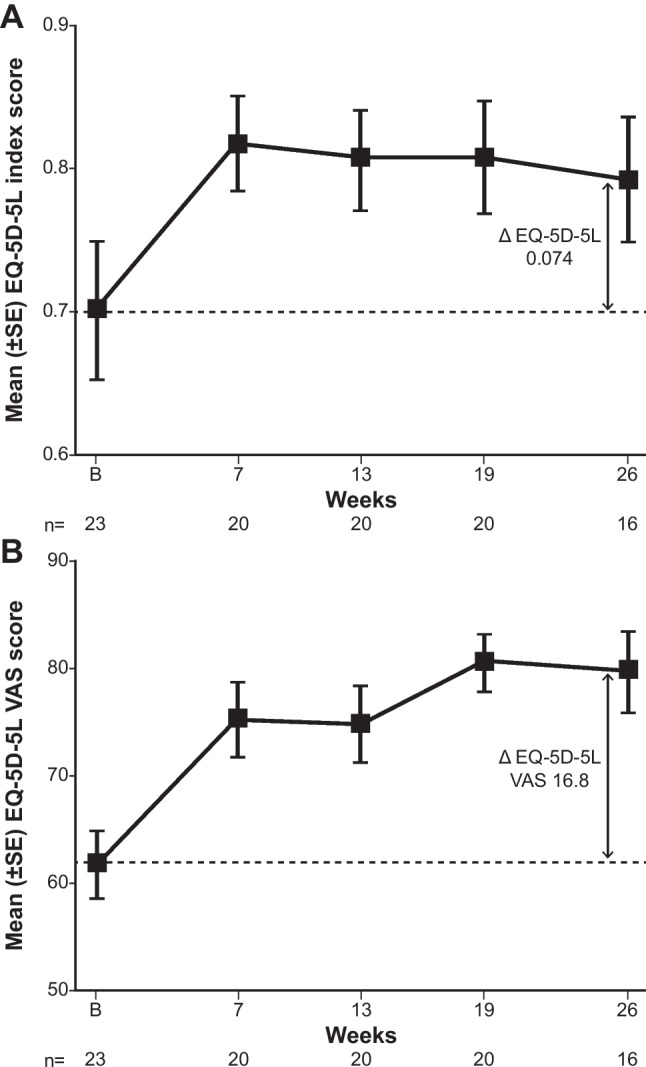
Mean change in EQ-5D-5L (**A**) index score and EQ-5D-5L VAS (**B**) from baseline to week 26. B, baseline; EQ-5D-5L, EuroQol 5-dimension 5-level questionnaire; EQ-5D-5L VAS, EuroQol 5-dimension 5-level questionnaire visual analogue scale; SE, standard error; VAS, visual analogue scale

**Fig. 4:**
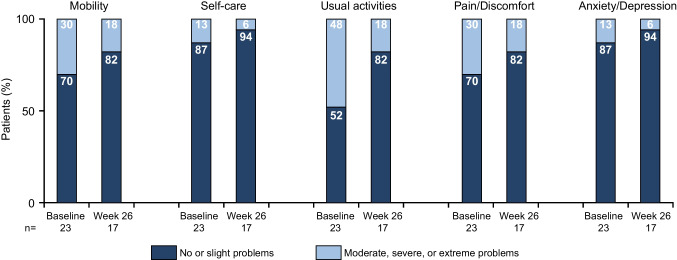
Proportion of patients reporting EQ-5D-5L domain responses as causing ‘no’ or ‘slight’ problems and ‘moderate’, ‘severe’, or ‘extreme’ problems. EQ-5D-5L, EuroQol 5-dimension 5-level questionnaire. Patients must select the extent to which their disease impacts each domain on a five-tier scale (from ‘no problem’ to ‘extreme problem’)^21^

Among 16 evaluable patients, mean (range) EQ-5D-5L index scores increased by 0.074 (−0.21 to 0.43) points from baseline to week 26 (Fig. 3A), while VAS scores increased by 16.8 (−10.0 to 45.0) in the same period (Fig. 3B). When analysed by response in each component of the EQ-5D-5L, the proportion of patients reporting ‘moderate, severe, or extreme’ (as opposed to ‘no or slight’) problems reduced from baseline to week 26 in all five domains after sutimlimab treatment (Fig. 4).

#### PGIC and PGIS

**Fig. 5:**
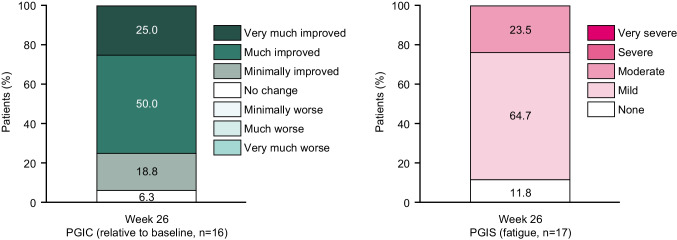
PGIC and PGIS fatigue scores at week 26. PGIC, Patient Global Impression of Change; PGIS, Patient Global Impression of Severity

At week 26, 94% of patients who completed the PGIC (*n*=16) indicated they felt that sutimlimab treatment had improved their disease (Fig. 5), and none felt that their disease had worsened from baseline (Supplementary Table 4).

At baseline, fatigue was reported by 83% of patients completing the PGIS, 67% of whom felt their fatigue to be moderate to severe (*n*=6) (Supplementary Table 5). At week 26, no patients reported severe fatigue and 89% of patients’ fatigue was reported as mild to moderate (Supplementary Table 5).

#### Incidence of CAD-related symptoms

At week 26, among patients with CAD-related symptoms, the incidence of haemoglobinuria and disabling circulatory symptoms were absent versus baseline, while incidence of acrocyanosis (21.1%) and Raynaud’s syndrome (10.5%) were similar versus baseline (Table 1). No major adverse vascular events were reported (including thrombosis).

#### Incidence of symptomatic anaemia

Improvement in anaemia symptoms from baseline to week 26 was reported; the incidence of fatigue decreased from 75.0 to 26.3%, weakness from 62.5 to 15.8%, shortness of breath from 54.2 to 15.8%, palpitations from 29.2 to 0.0% and chest pain from 8.3 to 5.3%.

## Discussion

Sutimlimab is a first-in-class, humanised, monoclonal antibody that selectively inhibits C1s and prevents classical CP activation, leaving alternative and lectin pathways intact [20].

In CARDINAL Part A, sutimlimab treatment resulted in rapid improvements in PROs among patients with CAD and a history of transfusion. The improvements in FACIT-Fatigue and the SF-12 were associated with a reduction in classical CP activity and increased total C4. This is the first study to report substantial improvements in PROs for patients receiving classical CP inhibition therapy for CAD.

Overall, least squares mean change of 11 points was observed from baseline to the TAT in FACIT-Fatigue score [4]. In a recent pooled analysis of data from CARDINAL and CADENZA, a FACIT-Fatigue score increase of 5 points was estimated to be a clinically important change in patients with CAD [21], which is consistent with estimates of between 2 and 10 points for other disease areas (e.g. rheumatoid arthritis, systemic lupus erythematosus [SLE], anaemia related to cancer) [3, 22–24].

In one patient who missed an infusion, withdrawal of sutimlimab for 28 days resulted in a FACIT-Fatigue score decline of 44%. Lost efficacy following withdrawal of treatment is supported by findings from a phase 1b study, where after rapid normalisation of bilirubin and haemoglobin following sutimlimab initiation, complement deposition on erythrocytes, haemolysis, and anaemia recurred within approximately 3–4 weeks of the final sutimlimab dose in all previous responders [25]. In responders who subsequently entered a named patient programme, retreatment with sutimlimab resulted in improvements in haemoglobin and bilirubin to the same extent as before the washout [25, 26].

SF-12 scores at study baseline were lower compared with the general US population 65–74 years of age [18], indicating reduced QOL in CAD. After 5 weeks of sutimlimab treatment, improvements in physical and mental domains coincided with near-complete classical CP activity inhibition and C4 normalisation, suggesting that classical CP activation and subsequent haemolysis may be key drivers of fatigue and poor QOL in patients with CAD. Improvements in EQ-5D-5L index and VAS scores also demonstrated rapid QOL improvement after initiation of sutimlimab. The greatest improvement was in the ‘usual activities’ domain, where the proportion of patients reporting moderate-to-extreme problems decreased by 30%, indicating improved QOL and emphasising the substantial contribution of fatigue to patients’ perception of general overall health.

A minimally clinically important difference of −0.011 to 0.158 has been reported for the EQ-5D-5L in a diverse range of conditions including diabetes mellitus, irritable bowel syndrome and chronic obstructive pulmonary disease [27, 28], while for the EQ-5D VAS, the minimum clinically important difference lies between 4.2 and 14.8 for Crohn’s disease [29]. Therefore, if we assume that minimum clinically important differences are similar in CAD, the mean EQ-5D-5L index and VAS score improvements of 0.074 and 16.8 observed in CARDINAL appear clinically meaningful. The PGIC and PGIS ratings both showed improvements at week 26. Additionally, among patients with CAD-related symptoms, the incidence of symptoms was reduced (haemoglobinuria and disabling circulatory symptoms) or remained generally unchanged (acrocyanosis, Raynaud’s syndrome) during the treatment period.

CAD should not be confused with cold agglutinin syndrome (CAS), a secondary form of cold haemolytic anaemia arising from an underlying condition such as infection, autoimmune disorder, or overt malignancy [30]. The distinction between CAD and CAS is important with PROs, as the patient experience relating to the underlying condition (e.g. SLE, infection, or malignancy) in CAS may differ substantially from that of patients with primary CAD, where such co-morbid conditions are absent [30]. In CAD, there is no clear underlying clinical condition eliciting additional fatigue, suggesting that it is more directly related to the inflammatory and haemolytic components of the disease. However, patients may suffer from co-morbidities that impact on QOL independently of anaemia and haemolysis and a considerable proportion of patients included in this cohort had at least one ongoing/resolved medical history. In the age group typical of patients with CAD [7], the likelihood of co-morbidities impacting patient QOL is elevated and their prevalence increases with age [31]. This may naturally exacerbate the disease burden, and patients with CAD with a higher Charlson Co-morbidity Index score have been observed to be at higher risk of thromboembolic events and associated fatigue and psychological distress [32, 33].

The observed improvements in PROs suggest that complement activity and haemolysis may play key roles in eliciting fatigue in patients with CAD. This is supported by a recent study [8] where classical CP activation enhanced proinflammatory cytokine expression, potentially contributing to fatigue beyond the impact of anaemia and consistent with observations of a proinflammatory state secondary to complement activation [5, 34]. In an in vitro study of *Escherichia coli-*induced inflammation, the release of proinflammatory cytokines (including interleukin [IL]-1, IL-6, IL-17, and tumour necrosis factor) was significantly reduced by a C1 inhibitor versus untreated human whole blood samples (*P*<0.05) [35]. Furthermore, a study of patients with chronic fatigue syndrome showed that 13 of 17 cytokines associated with fatigue severity (including IL-17) were proinflammatory [36]. These findings suggest fatigue may not be driven exclusively by anaemia in CAD and that by restricting the haemolytic and inflammatory components of fatigue, classical CP inhibition may reduce overall fatigue severity.

Prospective trials in CAD to date have not reported associations between an effect on markers of classical CP activity and PRO improvements. In a study of eculizumab (C5 terminal CP inhibitor) treatment in CAD, no significant improvements in QOL were reported [12]. However, in the current study, a close relationship was demonstrated between CP activation, haemolysis, haemoglobin increase and patients’ QOL. These were achieved using sutimlimab dosed at consistent intervals between infusions, with associated mild or moderate safety events [4].

Study limitations included the single-arm study design and small patient population (owing to the rarity of the disease). In addition, except for FACIT-Fatigue, general PRO measures were exploratory endpoints, and may not be sufficiently sensitive to detect differences in QOL specifically arising from the disease. Despite these encouraging results, the exploratory nature of these endpoints reflects that the small sample size did not provide sufficient power to detect statistical significance.

In conclusion, this study highlights that patients with CAD have consistently abnormal measures of QOL at baseline. Sutimlimab rapidly inhibits classical CP activity and subsequent haemolysis, which coincides with rapid, sustained improvements in fatigue symptoms and overall health-related QOL, further supporting the effectiveness of targeting upstream CP components in the management of patients with CAD.

### Supplementary Information

Below is the link to the electronic supplementary material.

Supplementary file1 (DOCX 141 KB)

## Data Availability

Qualified researchers may request access to patient-level data and related study documents, which may include clinical study report, study protocol with any amendments, blank case report form, statistical analysis plan, and dataset specifications. Patient-level data will be anonymised, and study documents will be redacted to protect the privacy of our trial participants. Further details on Sanofi’s data sharing criteria, eligible studies, and process for requesting access can be found at https://www.vivli.org/.
