# Correction to: Sutimlimab improves quality of life in patients with cold agglutinin disease: results of patient-reported outcomes from the CARDINAL study

**DOI:** 10.1007/s00277-024-05709-9

**Published:** 2024-03-23

**Authors:** Alexander Röth, Wilma Barcellini, Tor Henrik Anderson Tvedt, Yoshitaka Miyakawa, David J. Kuter, Jun Su, Xiaoyu Jiang, William Hobbs, Jaime Morales, Frank Shafer, Ilene C. Weitz

**Affiliations:** 1 Department of Hematology and Stem Cell Transplantation, West German Cancer Center, University Hospital Essen, University of Duisburg-Essen, Essen, Germany; 2 Fondazione IRCCS Ca’ Granda Ospedale Maggiore Policlinico, Milan, Italy; 3 Section for Hematology, Department of Medicine, Haukeland University Hospital, Bergen, Norway; 4 Department of General Internal Medicine, Saitama Medical University Hospital, Saitama, Japan; 5 Division of Hematology, Massachusetts General Hospital, Harvard Medical School, Boston, MA USA; 6 Sanof, Cambridge, MA USA; 7 Jane Anne Nohl Division of Hematology, Department of Medicine, University of Southern California – Keck School of Medicine, Los Angeles, CA USA

**Correction to: Annals of Hematology**

10.1007/s00277-022-04948-y

After publication of this paper, an error﻿ was made in the fourth paragraph of column 2 on page 2170, regarding the description of the possible score range of the SF-12 Mental Component Summary (MCS) and Physical Component Summary (PCS). In both places, the score range for the MCS and PCS was stated to be 0–100. These two measures are actually reported on a T-score metric, which has a mean of 50 and standard deviation (SD) of 10.

The text should be changed from: “(PCS and MCS, respectively; summary scores range 0–100)” to “(PCS and MCS scores are on a T-metric, with mean = 50 and SD = 10, referenced to the US general population)”.

The original article has been corrected.

