# Supplementary material for: Sutimlimab improves quality of life in patients with cold agglutinin disease: results of patient-reported outcomes from the CARDINAL study
Source: Ann Hematol. 2022 Aug 23;101(10):2169–77. doi: 10.1007/s00277-022-04948-y (PMC9463238; doi:10.1007/s00277-022-04948-y)
Supplement: Supplementary file 1 — Supplementary file1 (DOCX 141 KB) [file 277_2022_4948_MOESM1_ESM.docx]

**Supplementary Table 1** FACIT-Fatigue score at baseline and TAT among patients without haematologic response

|  | **FACIT-Fatigue score** | |
| --- | --- | --- |
|  | **Baseline** | **TAT** |
| Patient 1 | 41 | 42 |
| Patient 2 | 47 | 47 |
| Patient 3 | 38 | 24 |

FACIT, Functional Assessment of Chronic Illness Therapy; QOL, quality of life; TAT, treatment assessment timepoint (mean of Weeks 23, 25, and 26)

FACIT-Fatigue is a 13-item QOL assessment tool, wherein patients must evaluate the extent to which they feel fatigued by rating their agreement with the 13 items on a 5-point scale (0–4, from ‘not at all’ to ‘very much so’).^1^ The scores for each question are added together to give an overall value (maximum of 52). Items are reverse scored where appropriate, so that higher scores represent better functioning and less severe fatigue.^1^

Three patients did not have a clinically meaningful response; this was determined based on no improvement in haemoglobin, bilirubin levels that did not normalise, and/or little or minimal improvement in the FACIT-Fatigue shown above.

**Supplementary Table 2** Mean improvement from baseline in individual FACIT-Fatigue domain scores at Week 26

|  | **Mean (SEM) score at baseline** | **Mean (SEM) score at Week 26** | **Mean (SEM) score improvement from baseline to Week 26** |
| --- | --- | --- | --- |
| I feel fatigued | 1.86 (0.23) | 0.88 (0.23) | −1.00 (0.36) |
| I feel weak all over | 1.71 (0.26) | 0.53 (0.21) | −1.18 (0.37) |
| I feel listless | 1.24 (0.24) | 0.47 (0.17) | −0.94 (0.30) |
| I feel tired | 2.00 (0.24) | 0.94 (0.22) | −1.06 (0.33) |
| Trouble starting things | 1.52 (0.27) | 0.59 (0.17) | −0.94 (0.34) |
| Trouble finishing things | 1.48 (0.28) | 0.53 (0.17) | −0.94 (0.34) |
| I have energy | 1.71 (0.17) | 2.41 (0.21) | 0.71 (0.31) |
| Able to do my usual activities | 2.00 (0.28) | 2.65 (0.23) | 0.65 (0.36) |
| I need to sleep during the day | 1.57 (0.23) | 0.77 (0.20) | −0.88 (0.28) |
| I am too tired to eat | 0.33 (0.14) | 0.24 (0.14) | −0.18 (0.20) |
| Need help doing usual tasks | 1.05 (0.28) | 0.65 (0.26) | −0.59 (0.27) |
| Frustrated by being too tired | 1.33 (0.26) | 0.35 (0.15) | −0.82 (0.32) |
| Limit social activity, I am tired | 1.19 (0.27) | 0.59 (0.17) | −0.53 (0.37) |

QOL, quality of life; SEM, standard error of the mean.

### **Supplementary Table 3** Change in SF-12 component and subscale scores from baseline to Week 26

SF-12, 12-Item Short Form Health Survey.

|  | **Mean (SD) score at baseline (n=22)** | **Mean (SD) score at Week 26 (n=16)** | **Mean (SD) change from baseline to Week 26 (n=16)** |
| --- | --- | --- | --- |
| SF-12 component score **Physical component Mental component** | 38.7 (8.7)  49.8 (8.2) | 44.5 (8.6)  53.1 (8.2) | 5.4 (7.6)  4.4 (10.0) |
| SF-12 subscale scores **General health Physical functioning Role physical**  **Bodily pain Role emotional Vitality**  **Mental health Social functioning** | 39.4 (9.1)  38.8 (10.1)  38.6 (9.4)  49.9 (8.9)  45.7 (11.3)  41.5 (10.9)  52.5 (7.0)  44.0 (10.2) | 45.9 (8.4)  44.8 (11.1)  45.0 (9.2)  48.7 (9.3)  47.8 (8.7)  52.8 (7.1)  52.7 (7.8)  50.2 (6.1) | 5.5 (12.0)  6.4 (8.2)  7.1 (9.6)  −2.8 (9.7)  1.6 (10.5)  12.9 (12.3)  1.4 (9.0)  7.8 (10.7) |

**Supplementary Table 4** PGIC status from Weeks 5 to 26

| **PGIC status, n (%)** | **Week 5 (n=10)** | **Week 11 (n=11)** | **Week 17 (n=16)** | **Week 26 (n=16)** |
| --- | --- | --- | --- | --- |
| Much improved or very much improved | 8 (80) | 9 (82) | 9 (56) | 12 (75) |
| Minimally improved | 1 (10) | 1 (9) | 3 (19) | 8 (19) |
| No change | 1 (10) | 1 (9) | 2 (13) | 1 (6) |
| Minimally worse | 0 (0) | 0 (0) | 2 (13) | 0 (0) |
| Much worse or very much worse | 0 (0) | 0 (0) | 0 (0) | 0 (0) |

PGIC, Patient Global Impression of Change.

Percentages are based on the number of evaluable patients in the full analysis set.

**Supplementary Table 5** PGIS fatigue status from baseline to Week 26

| **PGIS fatigue status, n (%)** | **Baseline (n=6)** | **Week 5 (n=7)** | **Week 11 (n=10)** | **Week 17 (n=13)** | **Week 26 (n=17)** |
| --- | --- | --- | --- | --- | --- |
| None | 1 (17) | 0 (0.0) | 1 (10) | 3 (23) | 2 (12) |
| Mild | 1 (17) | 6 (86) | 4 (40) | 6 (46) | 11 (65) |
| Moderate | 2 (33) | 1 (14) | 4 (40) | 4 (31) | 4 (24) |
| Severe | 2 (33) | 0 (0) | 1 (10) | 0 (0) | 0 (0) |
| Very severe | 0 (0) | 0 (0) | 0 (0) | 0 (0) | 0 (0) |

PGIS, Patient Global Impression of Severity.

Percentages are based on the number of evaluable patients in the full analysis set.

**Supplementary Figure 1** Mean FACIT-Fatigue component scores from baseline through Week 26


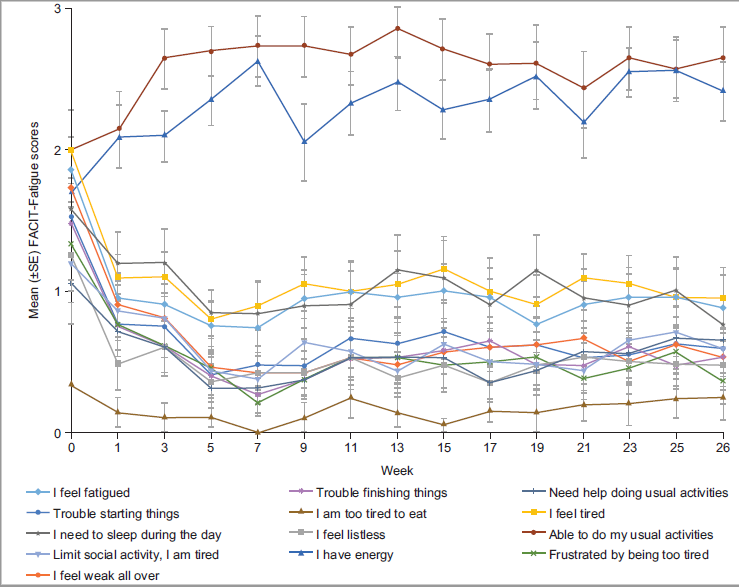


FACIT, Functional Assessment of Chronic Illness Therapy; SE, standard error.

FACIT-Fatigue is a 13-item QOL assessment tool, wherein patients must evaluate the extent to which they feel fatigued by rating their agreement with the 13 items on a 5-point scale (0–4, from ‘not at all’ to ‘very much so’).^1^ The scores for each question are added together to give an overall value (maximum of 52). Items are reverse scored where appropriate, so that higher scores represent better functioning and less severe fatigue.^1^

**Reference**

1. Acaster S, Dickerhoof R, DeBusk K, Bernard K, Strauss W, Allen LF (2015) Qualitative and quantitative validation of the FACIT-fatigue scale in iron deficiency anemia. Health Qual Life Outcomes 13:60. <https://doi.org/10.1186/s12955-015-0257-x>
